# Individual plasticity in response to rising sea temperatures contributes to an advancement in green turtle nesting phenology

**DOI:** 10.1098/rspb.2024.1809

**Published:** 2025-02-19

**Authors:** Mollie L. Rickwood, Eve Tucker, Damla Beton, Sophie Davey, Brendan J. Godley, Robin T. E. Snape, Erik Postma, Annette C. Broderick

**Affiliations:** ^1^ Centre for Ecology and Conservation, University of Exeter, Penryn Campus, Penryn, Cornwall TR10 9FE, UK; ^2^ Society for Protection of Turtles, Levent Daire 1, Ulus Sokak, Gönyelli, Nicosia, North Cyprus

**Keywords:** phenology, climate change, plasticity, adaptation, individual variation, demographic change

## Abstract

Phenological changes (i.e. shifts in the timing of biological events) are among the most frequently reported population-level responses to climate change and are often assumed to be adaptive and increase population viability. These may be driven by both individual-level phenotypic plasticity and population-level evolutionary and demographic changes. However, few studies have explored how individual-level versus population-level processes drive phenological trends. Using a 31-year dataset of over 600 individually marked nesting green turtles (*Chelonia mydas*), we quantify the population- and individual-level temporal trend in their first nest date. Of the latter, approximately 30% is attributable to individual phenological plasticity in response to sea surface temperature, with females advancing their nesting by 6.47 days for every degree (Celsius) increase. The remaining change is almost entirely explained by individual- and population-level changes in size and breeding experience (correlates of age), as well as the number of clutches laid per season. This is the first study of individual-level phenological change in a marine ectotherm, furthering our understanding of how this and similar species may respond to rising temperatures.

## 1. Introduction

Climate change increases extinction risk of many species, leading to unprecedented rates of biodiversity loss [1,2]. To accurately predict and mitigate further biodiversity declines, there is a need to understand if and how species and populations can adapt to future climatic regimes [3,4]. Changes in the phenology—the seasonal and interannual timing of biological events—are among the most frequently reported responses to climate change, and are often assumed to be adaptive [5–7]. Both evolution and individual plasticity could be important mechanisms driving phenological shifts, improving population persistence under environmental change [8,9]. In particular, individual phenotypic plasticity has been reported across a suite of traits and taxonomic groups in response to environmental change (see comprehensive reviews for invertebrates [10,11], reptiles and amphibians [12], fish [13], birds [14] and mammals [15]). This relies on long-term individual-based monitoring studies, which despite their tremendous value remain scarce and taxonomically biased [16]. However, the challenges associated with the individual-based monitoring of some taxonomic groups means that for many species we are limited to estimates of population-level changes in phenology (e.g. date of first reproductive event [17–19]). Hence, we are often unable to disentangle the roles of individual-level plasticity, demography and evolution in shaping phenological change [8,20].

Disentangling the contribution of individual-level (i.e. within-individual) versus population-level (i.e. among-individual) processes to trends in mean phenology is key if we are to understand the impact of environmental change, and the capacity of populations to adapt to these changes. While some studies have demonstrated individual phenotypic plasticity to be the predominant driver (e.g. in great tits (*Parus major*) [14] and bighorn sheep (*Ovis canadensis*) [21]), others have observed individuals to be consistent in their phenology (e.g. in Egyptian vultures (*Neophron percnopterus*) [22] and wood thrushes (*Hylocichla mustelina*) [23]). Thus, alternative mechanisms that change a population’s composition, including early-life, genetic and demographic changes have been proposed [24]. For example, Icelandic black-tailed godwits (*Limosa limosa islandica*) were found to be highly consistent in their arrival date, but new recruits arrived significantly earlier than their parents [25]. In contrast, within-individual increases in breeding experience were found to be the primary driver of an advancement in lay dates in Mandt’s black guillemot (*Cepphus grille mandtii*) [26]. These and other studies (e.g. [27,28]) demonstrate how the analysis of individual phenological variation can reveal the relative contribution of plasticity to long-term phenological trends [20,29–31].

Globally, migratory species are declining more rapidly than resident species [32], with examples in birds [33,34], terrestrial mammals [35], invertebrates [36] and freshwater fish [37]. These species are selected to time arrival at their breeding grounds to maximise their fitness, for example by optimizing nest site choice [38], increasing mating opportunity [39], maximizing food abundance [40] or enabling multiple or replacement clutches (e.g. [41]). Several factors can influence the timing of arrival, presenting a challenge for migratory species. First, the phenology and abundance of food sources at overwintering grounds and stop-over sites can influence arrival dates [42], with variation in food abundance shifting departure dates in several migratory birds [43,44]. Second, carry-over effects of environmental conditions at overwintering grounds and along migratory routes also influence reproductive phenology [33]. Finally, these decisions around migration timing are further complicated under climate change. For example, when species are cued by non-environmental factors such as photoperiod or when environmental change is proceeding at different rates between overwintering and breeding grounds [45], an issue that is particularly pronounced for long-distance migrants (versus short distance migrants) [46]. As such, climate change can result in asynchrony between the timing of arrival at the breeding grounds and the optimal conditions for reproduction, which may lead to reduced reproductive output, population declines and biodiversity loss [31,42,47,48].

Migratory marine species are some of the most endangered species globally, and among this group, sea turtles are the most threatened [49]. As ectotherms, the environmental temperature has a particularly profound impact on their reproductive biology, and thus they are especially vulnerable to climatic changes. In sea turtles, oviparity and the lack of parental care [50] make the nest environment—and primarily its temperature—a major determinant of embryonic development, sex determination and hatching success [51]. Accordingly, sea turtle populations display temporal patterns of nesting at rookeries that, at least historically, are aligned with seasonal temperature patterns, thereby ensuring optimal environmental conditions for egg development [52,53]. In addition to thermal niche tracking, herbivorous sea turtle species such as green turtles (*Chelonia mydas*) are sensitive to temperature-related changes in the abundance and distribution of marine algae and seagrass meadows, which may impact annual patterns at rookeries [54].

Although range shifts have been reported [55,56], the highly conserved nest site fidelity of sea turtles [57,58] may limit their ability to adapt to increasing temperatures through nest site choice. Similarly, their long generation times mean that maximum rates of evolutionary adaptation are likely too slow to keep pace with rates of environmental change [59]. Hence, phenotypic plasticity is most likely to be the primary mechanism for sea turtles to maintain a favourable climatic niche. Indeed, higher sea temperatures have been demonstrated to influence sea turtle nesting phenology [51], with earlier nesting consistently reported in loggerhead turtles (*Caretta caretta*) [17–19,60], yet delays to the onset of nesting in leatherback turtles (*Dermochelys coriacea*) [61]. While these studies provide strong evidence for an association between temperature and nesting phenology, in green turtles, all but one study found no effect [51]. Confirming if there is a role of individual plasticity in these observed trends may allow us to better understand the variation across species and populations.

Here, we address this knowledge gap through the analysis of three decades of individual-based data for the population of green turtles (*Chelonia mydas*) that nests on Alagadi Beach, North Cyprus. We first quantify the temporal trend in nesting phenology and sea surface temperature at the nesting beach (a proxy for the thermal environment experienced by breeding females), and the association between the two. We then decompose the temporal trend in phenology into a within- and an among-individual component. The former captures the effects of individual-level phenotypic plasticity and (correlates of) age. The latter captures the effect of changes in the composition of the breeding population due to evolutionary adaptation or early environmental effects, but also changes in, for example, the age or size structure of the population. Finally, we explicitly quantify the role of sea surface temperature, breeding experience and size in shaping these phenological trends by combining estimates of their effect on first nest date, and of how each has changed over time. Thereby, we provide a better understanding of the sensitivities and future responses of such species to climate change.

## 2. Methods

### (a) Study site and data collection

Data were collected on Alagadi Beach (35°33′ N, 33°47′ E), North Cyprus (electronic supplementary material, figure S1). Throughout the nesting season (mid-May to mid-August; 1992−2022), the beach was surveyed daily and nightly for nests. During night work, the beach was patrolled every 10 min between 20.30 and 05.30 to ensure all turtle activity was recorded. Nesting females are tagged post-oviposition with both flipper and passive integrated transponder (PIT) tags [62]. Their minimum curved carapace length (herein, size, measured in cm, from the anterior point at midline to the posterior notch at midline [63]) was calculated as the mean of three measurements taken at any given laying event.

The annual number of clutches increased from 55 in 1993 to 402 in 2022, reflecting the significant population recovery of green turtles in North Cyprus [64]. Females at this site lay, on average, 3–5 clutches every 2–4 years [65]. Across the study period, 3410 clutches were laid by 656 uniquely identified female turtles, and a further 347 clutches were not observed being laid and thus could not be assigned to individual females. Low and sporadic survey efforts meant the initial phase of nesting activity was not comprehensively recorded in 1992 and 2000. Survey effort was also reduced in 2020 and 2021 due to the COVID-19 pandemic. Although in these years we are confident that all females were captured as the peak of the nesting season was surveyed extensively, their first nests may have been missed. Hence, data from these years were omitted from population- and individual-level analyses of nesting phenology, but they were included when calculating the number of years (hereafter, breeding experience) in which a female had been recorded nesting. Data from 1993 and 1994 were included when calculating a female’s breeding experience (see below) but omitted from individual-level analyses of nesting phenology, as all females are unmarked at the start of the study and a reported remigration interval of 2–4 years [65] means that we cannot confidently distinguish between new recruits and returning breeders in these years. After excluding these 6 years and any clutches where the identity of the female was unknown, the dataset for individual-level analyses included 2849 clutches laid by 585 females. The median number of clutches laid by individual females was 2 (ranging from 1 to 6) per breeding season, with females breeding in a median of 2 seasons (ranging from 1 to 10) across the study. Overall, a median of 98.4% (*n* = 25) of clutches were attributed to individual females each year, varying from 75% in 1996 to 100% in 8 years of the study (electronic supplementary material, figure S3).

To capture variation in breeding experience, we counted the number of years (including the years of low survey effort) in which a female has been recorded nesting at Alagadi. Preliminary analyses showed that new recruits (first-time breeders) bred later than returning females, but that the first nest day remained relatively constant across subsequent breeding attempts (electronic supplementary material, figure S4). Breeding experience was therefore reduced to a variable with two levels: new recruits (coded as 0) and returning females that have completed at least one previous nesting season at Alagadi (coded as 1). Furthermore, we counted the number of clutches recorded for each female within a breeding season (*observed* clutch frequency). Internesting intervals (the number of days between nesting events) at Alagadi typically range between 10 and 15 days [66]. Thus, to account for missed nesting events where a female laid a clutch at another beach or was not encountered by the field team, we converted observed clutch frequency to *estimated* clutch frequency by assuming one, two or three clutches had been missed when internesting intervals were larger than 20, 30 and 45 days, respectively (following [66]).

Nesting date was measured as the day of the year with 1 January being day 1 and accounting for leap years. To enable comparison with previous work on sea turtle phenology (e.g. [17,19,60]), two metrics of population-level nesting phenology were considered: the earliest nest recorded each year (first nest date), and the population median nest date across all nests laid each year (*n* = 27). Here, the term ‘nest’ refers to a clutch being laid. The population-level nesting season duration was calculated for each year by subtracting the first nest date from the latest nest recorded each year (last nest date). Individual first nest date was calculated as the date of the earliest nest for each female within each season (*n* = 585).

### (b) Sea surface temperature data

Analyses were performed in R v. 4.3.0 [67]. We derived spatially averaged daily sea surface temperature for the waters adjacent to the nesting beach and four broadscale foraging areas previously identified by satellite tracking of individuals [68,69] using the package *terra* [70] (see electronic supplementary material, methods S1 for details). Using a sliding window approach implemented in the R package *climwin* [71], the mean sea surface temperature adjacent to the nesting beach between 29 March and 29 April was found to be the strongest predictor of nesting phenology (see electronic supplementary material, methods S1 for details). Hence, we used this temperature to estimate the effect of sea surface temperature.

### (c) Modelling nesting phenology

We first quantified temporal trends in the date of the first, median and last nest across all clutches laid within a year, as well as the nesting season duration and sea surface temperature, by regressing each against year (t) using separate linear regressions. Similarly, we estimated the relationship between sea surface temperature and annual first nest date, median nest date and nesting season duration.

**Figure 1.**
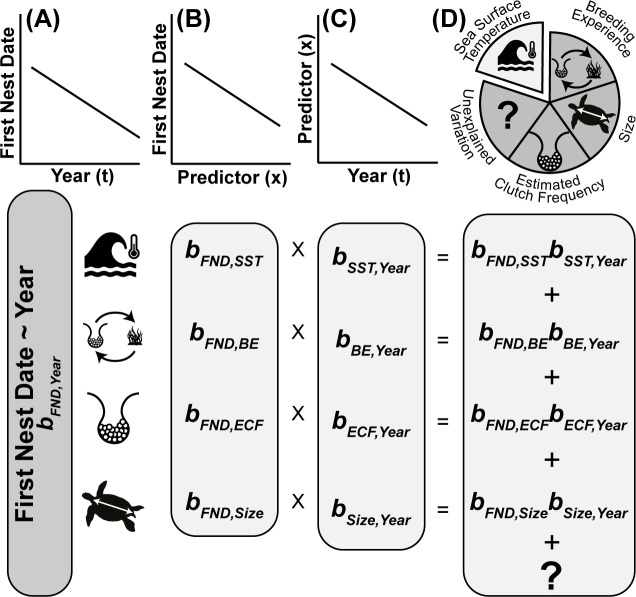
Quantifying the proportion of the individual- and population-level change in the first nest date explained by each predictor. (A) We first quantified how first nest date (FND) has changed over time, both within- (∆t) and among-individuals ($\overset{-}{t}$) (B). We then estimated the effect of each predictor (x) on the first nest date using a linear model in which first nest date is regressed against each predictor (∆x and $\overset{-}{x}$), as well as ∆t and $\overset{-}{t}$. (C) We quantified how each predictor (x) changed over time (t) on the within- and among-individual level by regressing ∆x and $\overset{-}{x}$ against ∆t and $\overset{-}{t}$, respectively. (D) Finally, we calculated the product of the slopes obtained in steps (B) and (C), which provides an estimate of the change in first nest date attributable to each predictor (x) (represented as a slice of the pie). The completed pie represents the sum of the effects of the predictors and any unexplained change in the first nest date over time within- and among-individuals, equal to (A). Abbreviations: FND, first nest date; SST, sea surface temperature; BE, breeding experience; ECF, estimated clutch frequency.

Using the *glmmTMB* R package [72], we then fitted a linear mixed model with individual first nest date as the response variable, year (t) as a fixed covariate and year of breeding and individual ID as categorical random effects. Thereby, this model provides an estimate of the annual change in mean individual-level first nest date (i.e. the mean of each individual’s first nest date; figure 1A) ($b_{FND,t}$).

We then used within-subject centring following van de Pol & Wright [73] to decompose this temporal trend into an among- and a within-individual component. To this end, we calculated the mean year ($\overset{-}{t}$) in which each female was observed breeding, and for each individual year in which she bred, the deviation from this mean (∆t). In the model outlined above, we then replaced t with both $\overset{-}{t}$ and ∆t as fixed covariates. The estimate for the effect of ∆t ($b_{FND,∆t}$) from this model provides us with the mean change per year in first nest date for an individual female (i.e. the within-individual change). The estimate for $\overset{-}{t}$ ($b_{FND,\overset{-}{t}}$) provides an estimate of the change in first nest date for a female that breeds *on average* 1 year later than another female (i.e. the among-individual change). If the observed rate of change in first nest date ($b_{FND,t}$) is solely attributable to within-individual changes in first nest date, estimates for $\overset{-}{t}$ and ∆t are identical. To test this, we fitted an additional model with $\overset{-}{t}$and t as covariates, where $b_{FND,t}$ now provides an estimate of the difference between the temporal trend observed within individuals ($b_{FND,\Delta t}-b_{FND,\overset{-}{t}}$) [73].

### (d) Decomposing temporal change in mean first nest date

We quantified how much of the within- and among-individual temporal trend could be attributed to changes in sea surface temperature, breeding experience, estimated clutch frequency and size, both within individuals and at the population level, by building on the approach outlined by Ellner *et al*. [74].

To this end, we included for each predictor (x, i.e. sea surface temperature, breeding experience, estimated clutch frequency or size) both the individual mean value ($\overset{-}{x}$) and the within-individual deviation from this mean (∆x) as additional covariates in the model outlined above. To facilitate model convergence, and to aid interpretation and visualization, individual mean predictors were all centred by subtracting the overall mean.

This extended model provides an estimate of the effect of within- and among-individual variation in each of these predictors on first nest date (figure 1B). For example, it provides an estimate of the change in first nest date if the sea surface temperature experienced by an individual increases 1°C ($b_{FND,\Delta SST}$), and if the mean sea surface temperature experienced by an individual goes up 1°C ($b_{FND,\;\bar{SST}}$). In this model, $b_{FND,\Delta t}$ and $b_{FND,\overset{-}{t}}$ capture the within- and among-individual temporal trend in first nest date not mediated by any of these predictors. Hence, it gives an indirect measure of the combined importance of sea surface temperature, estimated clutch frequency, breeding experience and size in shaping temporal trends in first nest date. In addition to these fixed covariates, this model again included the random effects of year and individual. See electronic supplementary material, figure S7 for diagnostic analyses and plots of model residuals, which did not indicate major violations of model assumptions.

Finally, we directly quantified the contribution of each predictor to the observed temporal change in first nest date following [74]. To this end, we quantified the temporal trend in each predictor (x) at both individual and population levels, by regressing ∆x against ∆t, and $\overset{-}{x}$ against $\overset{-}{t}$ in a series of simple linear regressions. For example, we regressed ∆SST against ∆t and $\bar{SST}$ against $\overset{-}{t}$ to obtain estimates of$\;b_{SST,\Delta t}$ and $\;b_{SST,\overset{-}{t}}$ (figure 1C). We then multiplied both estimates of the temporal change in x ($b_{∆x,\Delta t}$ and $b_{\overset{-}{x},\overset{-}{t}}$) with the estimates of their effects on first nest date ($b_{FND,\Delta x}$ and $b_{FND,\overset{-}{x}}$) to obtain the change in first nest date attributable to x ($b_{\Delta x,\Delta t}b_{FND,\Delta x}$), all else being equal. For example, the contribution of sea surface temperature to a change in first nest date within individuals is given by $b_{\Delta SST,\Delta t}b_{FND,\Delta SST}$. Doing this for each predictor hence provides a decomposition of the individual- and population-level temporal change in first nest date ($b_{FND,\Delta t}$ and $b_{FND,\overset{-}{t}}$, respectively) into the contributions of changes in sea surface temperature, breeding experience, estimated clutch frequency and size, and any change left unexplained (figure 1D). Note that to estimate the average increase in first nest date with a one-unit increase in ∆x and $\overset{-}{x}$ ($b_{FND,\Delta x}$ and $b_{FND,\overset{-}{x}}$, respectively), we used the model outlined above including ∆x and $\overset{-}{x}$ for each predictor, and ∆t and $\overset{-}{t}$, but this time without the random effects of year and individual. Although this model provides quantitatively very similar estimates, the partial contributions as calculated above do not add up exactly to the total temporal change if the random effects of year and individual are accounted for when estimating the effect of each predictor on first nest date if there is variation in the number of observations per year or individual.

## 3. Results

### (a) Population nesting phenology

**Figure 2.**
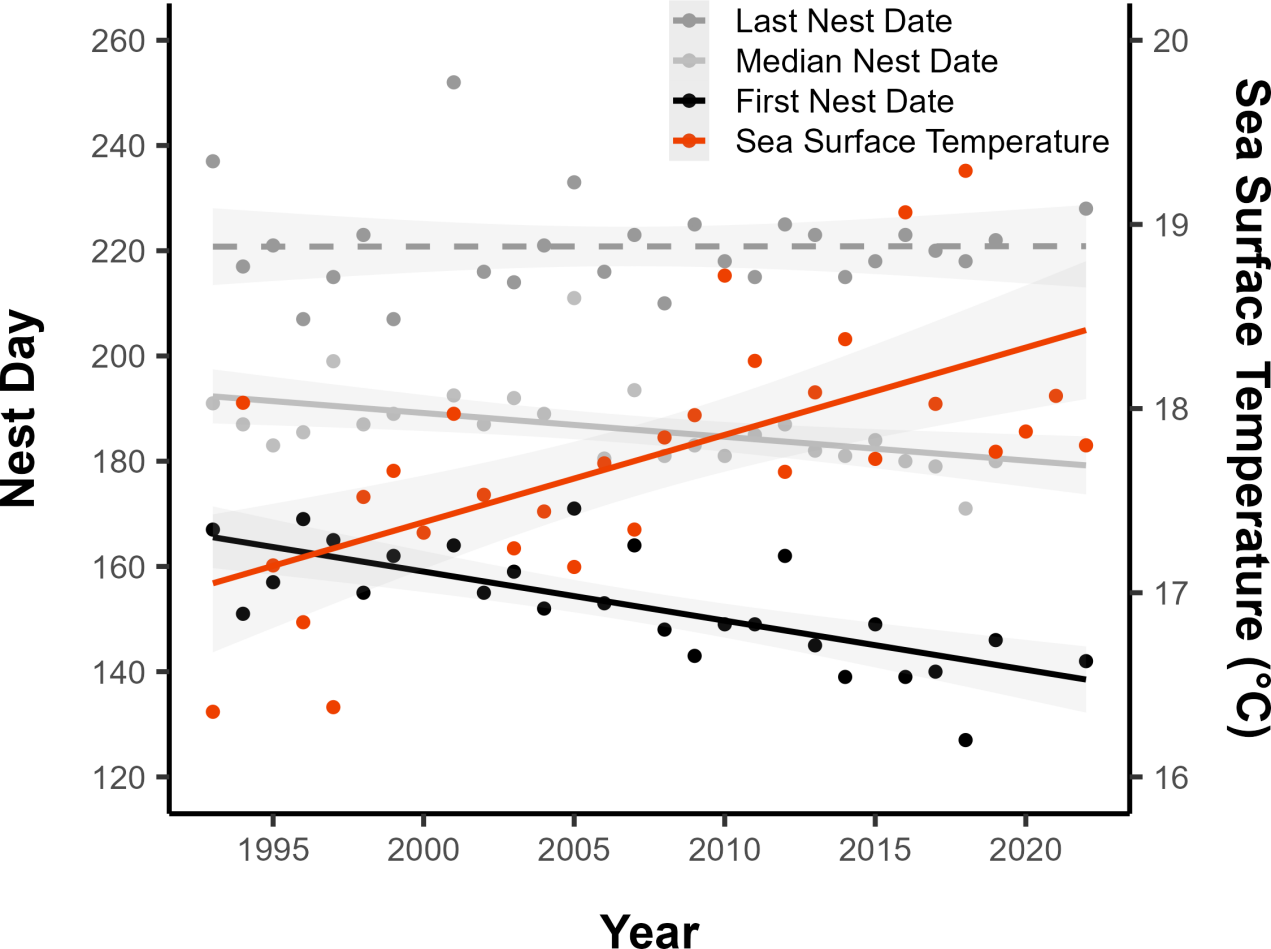
Temporal change in the annual nesting phenology of green turtles at Alagadi beach, North Cyprus. The trend in the population first, median and last nest date are represented by the black, light grey and dark grey lines, respectively. The trend in sea surface temperature is represented by the red line. Solid lines represent statistically significant trends, dashed lines represent non-significant trends and light grey shading represents the 95% confidence intervals.

Since 1993, the date of the earliest nest recorded each year (i.e. the annual first nest date for the population) has advanced by 0.93 d yr^−1^ (±0.18 s.e., *t*_25_ = −5.29, *p* < 0.001; figure 2) and the median nest date by 0.45 d yr^−1^ (±0.15 s.e., *t*_25_ = −2.92, *p* = 0.007; figure 2). The last nest date, however, has remained largely unchanged (*b* ± s.e. = 0.0028 ± 0.22 d yr^−1^, *t*_25_ = 0.01, *p* = 0.99; figure 2). This has resulted in an elongation of the nesting season, with the nesting season duration increasing by 0.94 d yr^−1^ (±0.24 s.e., *t*_25_ = 3.88, *p* < 0.001). Concurrently, the mean sea surface temperature has increased significantly (*b* ± s.e. = 0.05 ± 0.011°C yr^−1^, *t*_28_ = 4.39, *p* < 0.001; figure 2). An increase in sea surface temperature of 1°C coincides with an advancement of the population first nest date by 9.18 days (±2.25 s.e., *t*_24_ = −4.09, *p* < 0.001), the median nest date by 6.28 days (±2.22 s.e., *t*_24_ = −2.83, *p* = 0.009), and the nesting season duration increasing by 7.76 days (±3.67 s.e., *t*_24_ = 2.11, *p* = 0.045).

### (b) Decomposing individual and population responses in phenology

On average, individual first nest date has advanced by 0.61 d yr^−1^ (± 0.12 s.e., $\chi_{1}^{2}$ = 18.7, *p* < 0.001). This is the result of strong individual-level advancements in first nest date ($b_{FND,\Delta t}$ ± s.e. = −0.84 ± 0.14 d yr^−1^, $\chi_{1}^{2}$ = 27.8, *p* < 0.001), and a less pronounced advancement at the population level ($b_{FND,\overset{-}{t}}$ ± s.e. = −0.23 ± 0.15 d yr^−1^, $\chi_{1}^{2}$ = 2.4, *p* = 0.12; $b_{FND,\Delta t}-b_{FND,\overset{-}{t}}$ ± s.e. = 0.61 ± 0.14, $\chi_{1}^{2}$ = 19.6, *p* < 0.001).

There is a strong effect of sea surface temperature on first nest date at the individual level ($b_{FND,\Delta SST}$ ± s.e. = −6.47 ± 1.14 d °C^−1^, $\chi_{1}^{2}$ = 20.45, *p* < 0.001), whereas there is a smaller effect of sea surface temperature at the population level ($b_{FND,\bar{SST}}$ ± s.e. = −3.70 ± 1.41 d °C^−1^, $\chi_{1}^{2}$ = 6.57, *p* = 0.010). In addition to sea surface temperature, we find effects of breeding experience, estimated clutch frequency and size on first nest date. At the individual level, the first nest date advances by 3.75 days from a female’s first nesting year to subsequent breeding years (±1.03 s.e., $\chi_{1}^{2}$ = 13.09, *p* < 0.001). The effect of breeding experience on first nest date at the population level is stronger, with a population consisting of only experienced breeders nesting 5.92 days earlier than a population consisting solely of first-time breeders (±1.53 s.e., $\chi_{1}^{2}$ = 14.69, *p* < 0.001). Furthermore, there is an effect of estimated clutch frequency at the individual and population levels, with the first nest date advancing by 4.52 days (±0.40 s.e., $\chi_{1}^{2}$ = 112.70, *p* < 0.001) and 5.69 days (±0.40 s.e., $\chi_{1}^{2}$ = 170.51, *p* < 0.001), respectively, with each additional clutch laid. Finally, at the individual level, the first nest date advances by 0.41 days per cm increase in mean size (±0.23 s.e., $\chi_{1}^{2}$ = 2.99, *p* = 0.084). At the level of the population, however, larger females lay later ($b_{FND,\bar{Size}}$ ± s.e. = 0.24 ± 0.09 d cm^−1^, $\chi_{1}^{2}$ = 7.04, *p* = 0.008). This indicates that while an individual female nests earlier as it grows larger, a population of large females nests later than one composed of smaller females.

Decomposing the temporal trend in sea surface temperature into an individual- and population-level component reveals significant increases in sea surface temperature at both levels ($b_{\Delta SST,\Delta t}$ ± s.e. = 0.033 ± 0.0033°C yr^−1^, *t*_972_ = 9.87, *p* < 0.001; $b_{\bar{SST},\bar{t}}$ ± s.e. = 0.038 ± 0.0023°C yr^−1^, *t*_972_ = 16.45, *p* < 0.001; table 1; electronic supplementary material, figure S5A,B). Mean breeding experience (i.e. the proportion of returning females in a given year) and estimated clutch frequency have remained largely unchanged across the study period ($b_{BE,t}$ ± s.e. = −0.0031 ± 0.0038 breeding experience yr^−1^, *t*_23_ = −0.81, *p* = 0.42; $b_{ECF,t}$ ± s.e. = 0.011 ± 0.010 clutches yr^−1^, *t*_23_ = 1.08, *p* = 0.29; electronic supplementary material, figure S6B,C, respectively). However, female size has decreased ($b_{Size,t}$ ± s.e. = −0.17 ± 0.062 cm yr^−1^, *t*_23_ = −2.77, *p* = 0.011; electronic supplementary material, figure S6D). When decomposed into their individual- and population-level effects, we find opposing trends at both levels for each of these predictors. As we would expect, at the individual level, there was a positive effect of year on breeding experience ($b_{\Delta BE,\Delta t}$ ± s.e. = 0.058 ± 0.0026 breeding experience yr^−1^, *t*_972_ = 22.48, *p* < 0.001; table 1; electronic supplementary material, figure S5C), estimated clutch frequency ($b_{\Delta ECF,\Delta t}$ ± s.e. = 0.049 ± 0.0061 clutches yr^−1^, *t*_972_ = 7.98, *p* < 0.001; table 1; electronic supplementary material, figure S5E) and size ($b_{\Delta Size,\;\;\Delta t}$ ± s.e. = 0.021 ± 0.012 cm yr^−1^, *t*_972_ = 17.55, *p* < 0.001; table 1; electronic supplementary material, figure S5G), indicating that over time, a female gains breeding experience, and her estimated clutch frequency and size concurrently increase. However, at the population level, there is a decrease in mean breeding experience ($b_{\bar{BE},\overset{-}{t}}$ ± s.e. = −0.021 ± 0.0019 breeding experience yr^−1^, *t*_972_ = −10.87, *p* < 0.001; table 1; electronic supplementary material, figure S5D) and size ($b_{\bar{Size},\overset{-}{t}}$ ± s.e. = −0.27 ± 0.03 cm yr^−1^, *t*_972_ = −8.47, *p* < 0.001; table 1; electronic supplementary material, figure S5H), indicating that towards the end of the study period, the population is made up of less-experienced and smaller females. In contrast, there was a slight, non-significant decrease in the estimated clutch frequency across the study period ($b_{\bar{ECF},\overset{-}{t}}$ ± s.e. = −0.0098 ± 0.0071 clutches yr^−1^, *t*_972_ = −1.38, *p* = 0.17; table 1; electronic supplementary material, figure S5F).

**Table 1.** The contribution of each predictor (*x*) to the trend in first nest date across years (*t*), on (A) the individual level (Δ*x* and Δ*t*) and (B) the population level ($\overset{-}{x}$ and $\overset{-}{t}$). Predictors are sea surface temperature, breeding experience, estimated clutch frequency and size (i.e. minimum curved carapace length). The total individual- and population-level change in first nest date based on a model without other predictors ($b_{FND,∆t}$ and $b_{FND,\overset{-}{t}}$) are −0.79 and −0.24, respectively. FND, first nest date.

	parameter	**sea surface temperature**	**breeding experience**	**estimated clutch frequency**	size
(A)	effect of ∆t on ∆x: $b_{\Delta x,\Delta t}$	0.033	0.058	0.049	0.21
	effect of ∆x on first nest date: $b_{FND,\Delta x}$	−6.64	−3.75	−4.44	−0.45
	effect of ∆t on first nest date attributable to Δx: $b_{FND,\Delta x}b_{\Delta x,\Delta t}$	−0.22	−0.22	−0.22	−0.09
(B)	effect of $\overset{-}{t}$ on $\overset{-}{x}$: $b_{\;\overset{-}{x},\overset{-}{t}}$	0.038	−0.021	−0.0098	−0.27
	effect of $\overset{-}{x}$ on first nest date: $b_{FND,\overset{-}{x}}$	−4.10	−6.04	−5.60	0.31
	effect of $\overset{-}{t}$on first nest date attributable to $\overset{-}{x}$: $b_{FND,\overset{-}{x}}b_{\overset{-}{x},\overset{-}{t}}$	−0.16	0.13	0.055	−0.084

**Figure 3.**
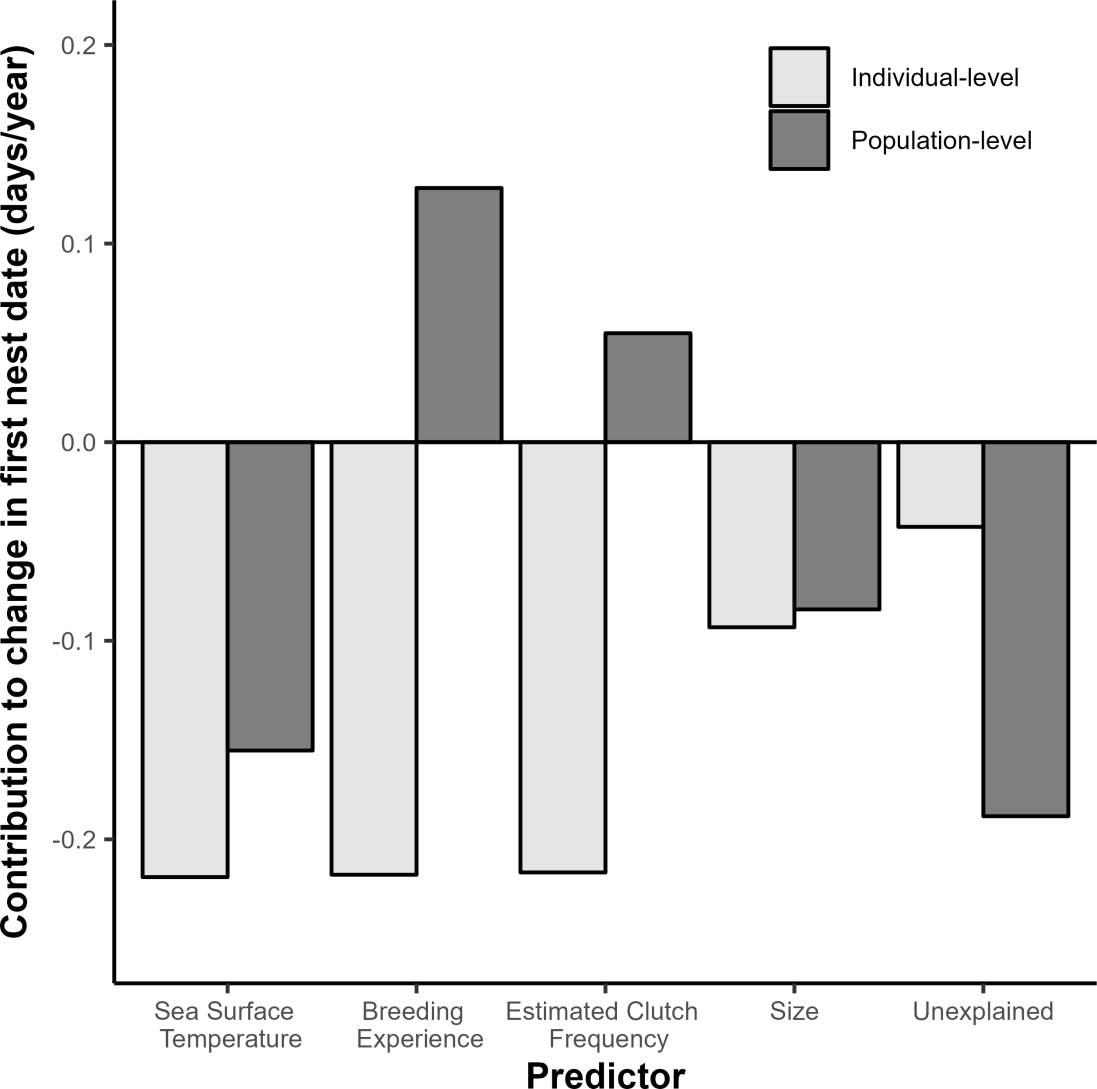
Contribution of individual- and population-level predictors to the change in first nest date of green turtles at Alagadi beach, North Cyprus. Light grey represents the individual-level effects and dark grey represents the population-level effects of sea surface temperature, breeding experience, estimated clutch frequency and size (i.e. minimum curved carapace length).

Figure 3 and table 1 show the contribution of the individual- and population-level effects of each predictor on the first nest date. The individual-level effects of sea surface temperature, breeding experience and estimated clutch freqency each account for around 28% of the individual-level advancements in first nest date, with size contributing another 12%, leaving 4% unaccounted for. However, sea surface temperature is the only predictor with a substantial contribution to the population-level shift in the first nest date, which is in line with sea surface temperature being the only predictor showing a substantial directional change across the study period (electronic supplementary material, figure S6).

## 4. Discussion

We demonstrate that individual plasticity to changing sea surface temperatures is a key driver of the phenological advancement of green turtle nesting at our study site, even after accounting for the confounding effects of breeding experience, estimated clutch frequency and size. Nonetheless, a female’s breeding experience and estimated clutch frequency explain a similar proportion of individual-level change in reproductive phenology. By contrast, at the level of the population, a change in sea surface temperature is the main driver of the advancement in nesting phenology, along with a small contribution from female size. However, population-level changes in breeding experience and estimated clutch frequency act to delay the first nest date, reducing the population-level advance. Thus, our study demonstrates the importance of considering the combined effects of individual- and population-level factors alongside environmental variables to understand the drivers of phenological change.

Studies consistently observe advances in spring/reproductive phenology across a wide range of taxa, including plants, insects, birds and mammals [75]. While reptilian studies are less common, warming-related advances in breeding phenology have been observed in freshwater turtles [76] and lizards [77,78], but the response in green turtles is less consistent [51]. In our study, we find a strong advance in nesting phenology, contradicting all but one [60] of the previous studies on green turtles that have observed no temperature-driven phenological change [54,79,80]. However, our study is the first to simultaneously examine the effects of non-environmental drivers at both individual and population levels. In doing so, we have revealed individual- and population-level mechanisms simultaneously shaping phenology, sometimes in opposite directions, which may explain the highly variable phenological responses among sea turtle populations and species.

It is possible that a physiological mechanism, such as the increased rate of egg development at higher temperatures [51,54,81], may underlie the plasticity we observed, with elevated local sea temperatures increasing rates of egg maturation, and thus earlier initiation of nesting following mating. However, plasticity in response to increasing sea temperature is not the sole driver of individual-level phenological variation, with a female’s breeding experience and breeding effort (i.e. estimated clutch frequency) accounting for similar proportions of an individual’s phenological advancement. Indeed, in several studies of long-distance migrant birds, phenological advancement with age and experience has been observed [82,83]. For example, in Cory’s shearwaters (*Calonectris borealis*), age and experience results in birds arriving earlier at breeding grounds as they advance their departure from non-breeding areas and reduce their migration time through optimization of migratory routes [84]. Similarly, in one of the only existing individual-level studies on a migratory marine mammal, the southern elephant seal (*Mirounga leonina*), experienced breeders advanced their breeding phenology with age [85]. Hence, a similar acquisition of knowledge and refinement of migratory behaviour may also explain our observations of age- and experience-related differences in nesting phenology.

Although we show plasticity to play an important role in shaping the observed phenological trend, this does not rule out any evolutionary change over the course of the study period [8]. Indeed, a substantial part of the population-level trend remains unexplained (figure 3). However, convincingly distinguishing between genetic and non-genetic changes in the population is challenging, and in line with this, conclusive evidence of adaptive evolution to climate change is rare in vertebrates [13,15,86]. In painted turtles (*Chrysemys picta*) that exhibit a plastic response in nesting date to temperature [76], the heritability of nesting phenology was found to be low to non-detectable [87]. In contrast, phenology was found to be moderately heritable in the southern elephant seal [85]. Furthermore, although phenology has been found to evolve in short-lived ectotherms such as chinook salmon (*Oncorhynchus tshawytscha*) [88], it is expected to be a very slow process in long-lived reptiles [89]. For example, whereas southern elephant seals may start reproducing after 4 years [90], green turtles are estimated to take over 30 years to reach sexual maturity [91]. On the whole, the contribution of genetic change across the time frame of our study is therefore most likely negligible.

A notable effect of changes in sea temperature is also observed at the population level, although a greater proportion of the population-level advancement remains unexplained. This indicates additional mechanisms also influence their nesting phenology. For example, although individual common terns (*Sterna hirundo*) exhibit repeatable migratory behaviour across years [92], birds arriving later than their conspecifics in their first breeding year continue to lay later throughout their lives [93]. In our population, a demographic behavioural change, not considered in our analysis, may influence population-level trends. Until 2010, almost 70% of green turtles nesting in Cyprus were known to forage in three discrete sites in Turkey and Libya [94]. However, subsequent stable isotope analysis and satellite tracking studies revealed the increasing importance of a foraging site at Lake Bardawil in Egypt, with the population increase being driven by the recruitment of females foraging at this site [68]. Thus, in the last 12 years of the study, an increasing proportion of females may have experienced different cues/timing of cues to initiate migration and were undertaking considerably shorter migrations [69]. This shift in foraging site is likely to be an important factor contributing to population-level phenology trends.

At the population level, an increase in the proportion of first-time breeders (electronic supplementary material, figure S5D) that, on average, nest later than older, more experienced females, is acting to delay nesting. In fact, it is the recruitment of higher numbers of naïve, inexperienced breeders that is driving the current population growth [64]. Furthermore, the decreasing female size across the study period is typical of a recovering population [95–97], indicating an increasing proportion of younger and likely smaller females (though size at sexual maturity is highly variable and not necessarily indicative of age [98,99]). As such, an accompanying decrease in estimated clutch frequency (ECF) would be expected, yet the ECF has remained largely unchanged. This may be the result of the elongation of the nesting season across the study period, increasing the opportunity for a greater number of clutches to be laid [89], and may offset the decrease in estimated clutch frequency associated with higher proportions of first-time breeders. Additionally, this may be compounded by the significant increase in sea temperature, as temperature-induced increases in metabolic rates may lead to a reduction in the internesting interval, allowing a greater number of clutches to be laid within a season [54,81,100,101].

As individual plasticity may be a critical determinant of the adaptive potential of a population [102], the temperature-dependent phenological plasticity of females at Alagadi indicates a comparatively positive outlook for this green turtle population. However, we did not examine whether the observed phenological advancement is sufficient to preserve the historic thermal niche for nesting, and to mitigate deleterious effects of climate change-related warming [20,103], such as reductions in hatching success and female-biased sex ratios observed in other sea turtle populations [104–106]. These effects may be particularly profound for lower latitude populations, many of whom are approaching/have reached their thermal limits [104,106]. However, as Alagadi beach sits at around 35°N and within a climate change hot-spot, the advancement in the first nest date observed here may be inadequate to preserve current thermal conditions across the nesting season, particularly as similar adaptive mechanisms in birds [107], the ectothermic brown anole (*Anolis sagrei*) [108] and freshwater turtles [76,109] have proved insufficient. Yet, as green turtle eggs have a comparatively high thermal tolerance [110], the required phenological shift to avoid climate debt (the time difference between the required and observed phenological change) may be lower for this species. Nevertheless, as ectotherms whose physiology is highly dependent on their environmental temperature [111], sea turtles are under strong selection to occupy their optimal niche for nesting if it exists, whether that requires phenological and/or spatial adaptation.

## 5. Conclusions

This study is the first to demonstrate that individual plasticity to rising sea surface temperatures contributes to an advancement in reproductive phenology of a migratory marine ectotherm. In addition, by accounting for the individual- and population-level effects of breeding experience, estimated clutch frequency and female size, we explain almost all the variation in nesting phenology of green turtles at our study site. While we find that individual plasticity to environmental cues may be a key phenological driver for long-lived marine ectotherms [89], whether this is sufficient to mitigate the increased thermal stress from climate warming warrants further study. To this end, future studies should consider the physiological, geographic and demographic factors shaping both phenology and reproductive success and survival at both individual and population levels to truly understand the role individual plasticity plays in the face of an ever-changing climate.

## Data Availability

The code and data supporting the findings of this study are available at [112]. Sea surface temperature data were derived from the following resource available in the public domain: EU Copernicus Marine Service Information [113]. Supplementary material is available online [114].
